# Ultra-Short-Term Offshore Wind Power Prediction Based on PCA-SSA-VMD and BiLSTM

**DOI:** 10.3390/s24020444

**Published:** 2024-01-11

**Authors:** Zhen Wang, Youwei Ying, Lei Kou, Wende Ke, Junhe Wan, Zhen Yu, Hailin Liu, Fangfang Zhang

**Affiliations:** 1 Institute of Oceanographic Instrumentation, Qilu University of Technology (Shandong Academy of Sciences), Qingdao 266075, China; wangzqd@qlu.edu.cn (Z.W.); yingyw@stu.qlu.edu.cn (Y.Y.); wan_junhe@qlu.edu.cn (J.W.); yuzhen8907@qlu.edu.cn (Z.Y.); qdliuhailin@aliyun.com (H.L.); zhff4u@qlu.edu.cn (F.Z.); 2 Department of Mechanical and Energy Engineering, Southern University of Science and Technology, Shenzhen 518055, China; kewd@sustech.edu.cn

**Keywords:** offshore wind farm, power prediction, variational modal decomposition, sparrow algorithm, long- and short-term memory neural networks

## Abstract

In order to realize the economic dispatch and safety stability of offshore wind farms, and to address the problems of strong randomness and strong time correlation in offshore wind power forecasting, this paper proposes a combined model of principal component analysis (PCA), sparrow algorithm (SSA), variational modal decomposition (VMD), and bidirectional long- and short-term memory neural network (BiLSTM). Firstly, the multivariate time series data were screened using the principal component analysis algorithm (PCA) to reduce the data dimensionality. Secondly, the variable modal decomposition (VMD) optimized by the SSA algorithm was applied to adaptively decompose the wind power time series data into a collection of different frequency components to eliminate the noise signals in the original data; on this basis, the hyperparameters of the BiLSTM model were optimized by integrating SSA algorithm, and the final power prediction value was obtained. Ultimately, the verification was conducted through simulation experiments; the results show that the model proposed in this paper effectively improves the prediction accuracy and verifies the effectiveness of the prediction model.

## 1. Introduction

With the continuous promotion of the dual-carbon target and the requirement for high-quality economic development, China’s power system is undergoing a transformation towards a new type of power system based on renewable energy [1]. One of the most important forms of renewable energy is wind energy, which is highly favored by countries worldwide due to its non-polluting nature and unrestricted availability. As a result, the wind power industry is experiencing rapid development [2,3]. However, wind farm output is significantly influenced by environmental conditions. In comparison to onshore wind farms, offshore wind farms face rapidly evolving meteorological conditions and complex sea states with intermittent, volatile, and uncertain characteristics. While offshore wind farms provide clean energy to meet human needs, they also present new challenges for the safe and stable operation of the offshore power system [4,5]. Therefore, the development of a precise wind-power-forecasting model is crucial. Through comprehensive analysis of historical data using data mining techniques or machine learning algorithms, it is possible to extract pivotal information and trends inherent in the data. Such insights are instrumental in guaranteeing the economic viability, safety, and stability of offshore wind farms, thereby playing a vital role in the successful integration of wind power into the grid [6,7].

Wind power forecasting can be categorized into long-term, medium-term, short-term, and ultra-short-term according to the time scale. Among them, the ultra-short-term prediction can provide the prediction of wind power in the next zero to four hours, which is highly correlated with the operation of the power generation system integrated in the wind farm, and by monitoring the wind-power-prediction data in real time, abnormalities of the wind turbines can be found in time to reduce the loss of equipment failures and the cost of maintenance. Therefore, designing an accurate, fast, and reliable ultra-short-term wind-power-prediction method is an important factor to improve the accuracy of forecasting for ocean wind power generation [8].

Current wind-power-forecasting methods can be broadly categorized into physical modeling methods, statistical analysis methods, and machine learning methods [9,10]. Physical modeling methods need to consider many factors, such as the marine environment, meteorological conditions, equipment characteristics, and so on [11]. Since such complex models require a large number of computational resources and data support, as well as the modeling and processing of multiple uncertainties, physical modeling approaches to deal with offshore wind power prediction have many difficulties and challenges [12]. Statistical analysis methods are used to predict wind power by uncovering the inter-relationships between data, such as wind speed, wind direction, humidity, and wind power in wind farms. In short-term forecasting, statistical models usually show more accurate results than physical methods [13], including commonly used methods such as (Autoregression Integrate Moving Average, ARIMA) [14]. The machine learning method builds a model based on the historical data of wind power, obtains certain laws by training the model, and applies the derived laws to predict future data.

The emergence of new technologies such as artificial intelligence and big data technology, such as artificial neural networks (ANNs) [15], Markov chain (MC) [16], extreme learning machine (ELM) [17], random forests (RFs) [18], and long–short-term memory neural network (LSTM) [19], has provided a new impetus for the application of the field of wind-power-prediction systems [20]. The literature [21] compares LSTM with other prediction models, and the results show that the LSTM model outperforms other prediction models in both long-term and short-term prediction. As an extension of LSTM, the bidirectional long- and short-term memory neural network (BiLSTM) was shown to achieve higher wind-power-prediction accuracy compared to LSTM [22]. However, BiLSTM needs to achieve its expected results with a high degree of accuracy and relevance due to the problems of model complexity and long training time [23]. Therefore, the introduction of dimensionality reduction algorithms in predictive models can improve the computational efficiency of the models. Principal component analysis (PCA) is an effective method for data dimensionality reduction by analyzing the covariance structure of multivariate data series, calculating the contribution of each series, and selecting the primary series to be expressed. The literature [24] uses the PCA-BP method to filter the input data of the model, eliminating the information with low redundancy and weak correlation in the data, which reduces the complexity of the model and improves the efficiency and accuracy of the subsequent prediction model.

Offshore wind-power time-series data samples display inherent non-linear and unstable traits, primarily due to native properties and the impact brought about through the offshore wind field environment. The objective of data decomposition techniques is to partition an original, non-stationary, volatile, and insignificantly regular time series. This partition is conducted to attain several stable subsequences, thereby potentially enhancing the model’s precision. The commonly used signal decomposition techniques include wavelet decomposition (wavelet) [25], empirical modal decomposition (EMD) [26], ensemble empirical modal decomposition (EEMD) [27], and variational modal decomposition (VMD) [28], etc. EMD and EEMD can be useful in dealing with offshore wind data because of the inclusion of extremely large eigen timescales or the existence of similar eigen timescales, which cause two neighboring IMF waveforms to overlap, resulting in a certain degree of modal aliasing and spurious components [29]. The variational modal decomposition (VMD) can fit the adaptive frequency and bandwidth of each component to the original signal, which can effectively avoid the problem of modal aliasing in each component and has been widely applied in the field of wind power prediction [30]. The literature [31] experimentally confirmed that the VMD decomposition can overcome the modal overlapping problem and is more stable than the traditional signal decomposition method.

Although numerous studies have addressed wind power prediction using data decomposition and reconstruction methods, there is always a lack of stable and efficient methods for determining the total number of modal decompositions, k, and the quadratic penalty coefficient, α, in decomposing the wind power sequence signals using the VMD, and the selection of these parameters will directly affect the final effect of decomposition. The way of relying on the human setting of these parameters is subjective and may affect the accuracy of power signal decomposition. The introduction of intelligent optimization algorithms can overcome the shortcomings of the above methods in which the parameters need to be determined manually, and the automatic optimization search of the parameters can be achieved by intelligent optimization algorithms. Commonly used algorithms include Particle Swarm Optimization (PSO) [32], Genetic Algorithm (GA) [33], Whale Optimization Algorithm (WOA) [34,35], Firefly Algorithm(FA) [36], sparrow search algorithm (SSA) [37], and so on. The literature [38] proposed a VMD-SSA-LSTM algorithm. The power load data is decomposed into intrinsic modal functions with different characteristics and frequencies using VMD, and then the processed data is used to train the LSTM model with the help of the sparrow search algorithm, which can be effectively applied to short-term power load forecasting. The literature [39] applies the Whale Optimization Algorithm for automatic optimization of the core parameters of VMD (K value and penalty coefficient α). The improved sparrow search algorithm SSA is introduced to optimize the learning parameters in the least-squares support vector machine LSSVM, and the combined model in this paper has greater improvement in prediction accuracy than the existing single prediction model and the common combined model.

In summary, this paper proposes an ultra-short-term offshore-wind-power-prediction method based on PCA-SSA-VMD-BiLSTM. The main innovations of this paper are as follows.

(1) The PCA method is used to downscale the multidimensional variables of wind power so as to effectively reduce data redundancy and improve the model prediction efficiency.
(2) On the basis of SSA and VMD, an improved decomposition method is proposed, which adopts SSA adaptive optimization parameters, i.e., modal decomposition number and penalty coefficients, in order to optimize the decomposition effect and improve the quality of inputs to the prediction model.
(3) The BiLSTM neural network is optimized using the sparrow search algorithm to search the global optimal solution more efficiently for the parameters, such as the initial learning rate and the number of hidden units in BiLSTM to improve the accuracy of the model.

This article is organized as follows. Section 2 describes the models and principles used in this paper, while Section 3 presents the data used and the comprehensive experimental analysis and evaluation. Conclusions and potential future work are described in Section 4.

## 2. Methods and Models

The purpose of this section is to briefly introduce the methods used in this study, including the principal component analysis (PCA) method, the sparrow search algorithm (SSA), the Variational Modal Decomposition (VMD) algorithm, and the Bidirectional Long- and Short-Term Memory Neural Network (BiLSTM) and the PCA-SSA-VMD-BiLSTM models.

### 2.1. PCA Data Downscaling

The principal component analysis (PCA) method is extensively utilized for the reduction in feature vectors, thereby facilitating the dimensionality reduction in data. This reduction streamlines the computations handled by the neural network, resulting in an enhanced computational speed. The idea of PCA is to construct new variables using linear combinations of the original variables, which reflect as much information as possible about the original variables without correlation. It maps n-dimensional features to a k-dimensional space (k < n), which are completely new orthogonal features, i.e., principal components. Principal components are reconstructed k-dimensional features, not just other n-k dimensional features removed from n-dimensional features. Each new feature has its own unique meaning, and the information about the data is mainly reflected in the variance. Features with large variance reflect the fact that the main information is concentrated in the original multiple variables. Cumulative variance contribution rate is usually used as a measure. Dimensions with cumulative variance contribution greater than or equal to 85% are usually selected as the reference scale for PCA downscaling.

Assuming a sample dataset $X=\{x_{11},x_{12},x_{ij},\ldots ,x_{mn}\}$ , *i* is the time node and *j* is the environmental factor of the dataset.

Step 1: Normalize the data, unified data dimension, *X′*

(1) $$X^{\prime }=\frac{x-x_{\min}}{x_{\max}-x_{\min}}$$

Step 2: The matrix is linearly transformed to obtain the covariance matrix, *R*:

(2) $$R=\frac{1}{n}{\left(X^{*}\right)}^{T}X^{\prime }$$

Step 3: Characteristic matrix is obtained by solving |λI−R|=0 , variance contribution rate $\eta_{i}$ and cumulative contribution rate $\eta_{\Sigma }(p)$ are, respectively,

(3) $$\eta_{i}=\frac{100\%\lambda_{i}}{\sum_{i=1}^{p}\lambda_{i}}$$

(4) $$\eta_{\Sigma }(p)=\sum_{i=1}^{p}\eta_{i}$$

Take the eigenvectors corresponding to the first p eigenvalues to form the coordinate system $V_{p}=\left(v_{1},v_{2},\cdots ,v_{p}\right)$ after dimensionality reduction; that is the solution of principal component analysis.

### 2.2. Sparrow Search Algorithm

The sparrow search algorithm (SSA) is a population-based intelligence algorithm, inspired by the foraging and anti-predator behaviors of sparrows. It is distinguished by its robust optimization capabilities, rapid convergence, and high stability. In this study, SSA is employed in data processing and deep learning to optimize the hyperparameters of Variational Mode Decomposition (VMD) and Bidirectional Long Short-Term Memory (BiLSTM) networks. Within SSA, discoverers with superior fitness values are given precedence in accessing food resources during the search process. Additionally, the discoverer plays a crucial role in sourcing food for the entire sparrow population and guiding the foraging paths for all joiners, resulting in a broader search range for the discoverer. The method for updating the discoverer’s location in each iteration is delineated as follows:

(5) $$X_{i,j}^{t+1}=\left\{\begin{array}{l} X_{i,j}^{t}\exp\left(-\frac{i}{\alpha Iter_{\max}}\right),\;R_{2}<S_{T}\\ X_{i,j}^{t}+\varphi L,\;R_{2}\geq S_{T} \end{array}\right.$$

*T* denotes the number of iterations, and j denotes the dimension. $Iter_{\max}$ is the maximum number of iterations. $X_{i,j}$ denotes the location information of the *i*th sparrow in the jth dimension. *α* is a random number in (0, 1]. *φ* is a random number obeying normal distribution. $R_{2}<S_{T}$ indicates that it is a safe environment at the present time. If $R_{2}\geq S_{T}$ , it indicates that a predator has appeared at this time and all sparrows need to move to a safe point.

The position of the joiners is updated accordingly:

(6) $$X_{i,j}^{t+1}=\left\{\begin{array}{c} \varphi \exp\left(\frac{X_{\mathrm{worse}}^{t}-X_{i,j}^{t}}{i^{2}}\right),\;i<n/2\\ X_{P}^{t+1}+\left|X_{i,j}^{t}-X_{P}^{t+1}\right|A^{+}L,\;\mathrm{other} \end{array}\right.$$

$X_{P}$ denotes the optimal position belonging to the discoverer, and $X_{\mathrm{worst}}$ denotes the global worst position. a denotes a 1 × D matrix, where each element of the matrix is a random 1 or −1. *i* > *n*/2, the *i*th accession with a low level of adaptation belongs to the starving state, and in order to obtain energy it needs to fly elsewhere to forage for food.

Assuming that the sparrows who are aware of the danger make up 10 to 20 percent of the total, and that the initial positions of these sparrows are randomly generated, the mathematical expression is as follows:

(7) $$X_{i,j}^{t+1}=\left\{\begin{array}{c} X_{\mathrm{best}}^{t}+\beta \left|X_{i,j}^{t}-X_{\mathrm{best}}^{t}\right|,\;f_{i}>f_{g}\\ X_{i,j}^{t}+K\left(\frac{\left|X_{i,j}^{t}\;-\;X_{\mathrm{worse}}^{t}\right|}{\left(f_{i}\;-\;f_{w}\right)\;+\;\gamma }\right),\;f_{i}=f_{g} \end{array}\right.$$

$X_{\mathrm{best}}$ denotes the global optimal position, and *β* is a step control parameter for a normal distribution with mean 0 and variance 1. *K* is a random number in [−1, 1], $\;f_{i}$ is the current individual fitness value. $f_{g}$ and $f_{w}$ denote the current best and worst fitness values. $\;f_{i}$ > $f_{g}$ denotes that the sparrow is very vulnerable to predators. $X_{\mathrm{best}}$ indicates the current safest position of the sparrow at this time. When $\;f_{i}$ = $f_{g}$ , it means that the sparrow is in danger and needs to move closer to other sparrows to reduce the risk of being attacked by predators. *K* denotes the direction of movement of the sparrow. *γ* is a constant, in order to avoid 0 in the denominator.

### 2.3. Variational Modal Decomposition

VMD is a variational estimation method to decompose non-linear signals by multiresolution, which belongs to a completely non-recursive model, and it determines the IMF by iteratively searching for the optimal solution of the variational model in the process of obtaining decomposed components, so as to be able to adaptively realize the frequency dissections of the signal data, as well as the effective separation between the components. In this paper, the power sequence is decomposed by VMD pre-processing after SSA optimization to obtain multiple modal functions with different frequency characteristics.

Firstly, the variational problem is constructed, assuming that the original time series signal *S* is decomposed into *K* components μ, ensuring that the decomposed sequence is a modal component with finite bandwidth having a center frequency, and at the same time, the sum of the estimated bandwidths of each modality is minimized, and the constraint is that the sum of all the modalities is equal to that of the original signal, then the corresponding constrained variational expression is as follows:

(8) $$\min_{\left\{u_{k}\right\},\left\{\omega_{k}\right\}}\left\{\sum_{k=1}^{K}{\left\|\partial_{t}\left[\left(\delta (t)+\frac{j}{\pi t}\right)*u_{k}(t)\right]e^{-j\omega_{k}t}\right\|}_{2}^{2}\right\}$$

(9) $$s.t.\;\sum_{k=1}^{K}u_{k}=S$$

In the formula, $\left\{u_{k}\right\}$ denotes the kth mode; $\left\{\omega_{k}\right\}$ is the set of center frequencies of all modes; k is the number of functions of all modes, and $\partial_{t}$ is the Dirac distribution;

Solving the constrained variational expression and introducing the penalty parameter α, Lagrange multiplication operator *λ* transforms the constrained variational problem into an unconstrained variational problem, obtaining the augmented Lagrange expression as follows:

(10) $$\begin{array}{l} \begin{array}{l} L\left(\left\{u_{k}\right\},\left\{w_{k}\right\},\lambda (t)\right)\\ =\alpha \sum_{k}{\left\|\partial_{t}\left[\left(\delta (t)+\frac{j}{\pi t}\right)*u_{k}(t)\right]e^{-jwt}\right\|}_{2}^{2} \end{array}\\ \;+{\left\|f(t)-\sum_{k}u_{k}(t)\right\|}_{2}^{2}+\left(\lambda (t),f(t)-\sum_{k}u_{k}(t)\right) \end{array}$$

In the formula, $\left\{u_{k}\right\}$ denotes each intrinsic mode function (IMF) component after decomposition; $\left\{\omega_{k}\right\}$ denotes the center frequency of each component; α is the quadratic penalty factor; *λ* is the Lagrange multiplier; $\partial_{t}$ denotes the partial derivative of the function at time *t*; and * denotes the convolution operator.

The alternating direction multiplier method of alternating updates is used to transform the above medium variational problem into an alternative model. The updated formulas for $u_{k}$ and $\omega_{k}$ are as follows:

(11) $${\hat{\mu }}_{k}^{n+1}(\omega )=\frac{f(\omega )-\sum_{i\neq k}{\hat{u}}_{i}^{n}(\omega )+\frac{{\hat{\lambda }}^{n}(\omega )}{2}}{1+2\alpha {\left(\omega -\omega_{n}^{k}\right)}^{2}}$$

(12) $$\omega_{k}^{n+1}=\frac{\int_{0}^{\infty }\omega \left|{\hat{\mu }}_{k}^{\hat{n}+1}{\left(\omega \right)}^{2}\right|d\omega }{\int_{0}^{\infty }\left|{\hat{\mu }}_{k}^{\hat{n}+1}{\left(\omega \right)}^{2}\right|d\omega }$$

In the formula, $\hat{f}(\omega )$ , ${\hat{\mu }}_{i}(\omega )$ , $\hat{\lambda }(\omega )$ , and ${\hat{\mu }}_{k}^{\hat{n}+1}(\omega )$ denote the Fourier transforms of f(t) , μ(t) , λ(t) , and $\mu_{k}^{\hat{n}+1}(t)$ , respectively. The k narrowband IMF components after decomposition of the original sequence of wind power are obtained by the Fourier inverse transform, so that the original signal of wind power is adaptively segmented in the frequency domain.

### 2.4. BiLSTM Neural Network

**Figure 1:**
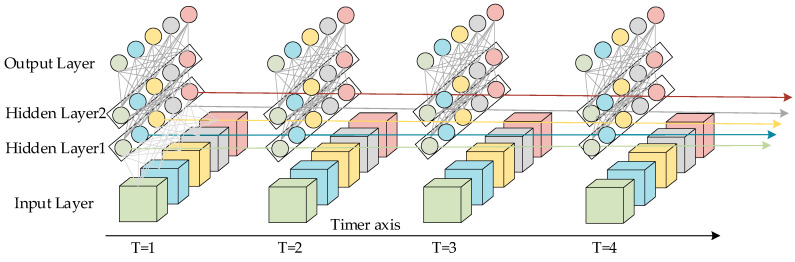
Spatial structure of LSTM model.

**Figure 2:**
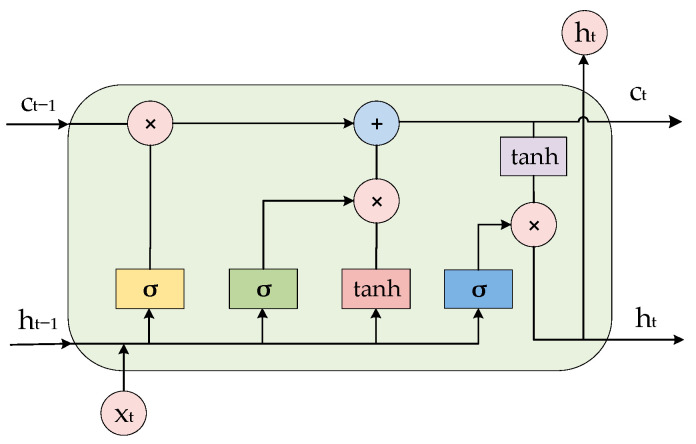
Internal structure of LSTM model.

LSTM is a recurrent neural network model with the ability to memorize long- and short-term information improved on the basis of recurrent neural network RNN model. The structure of LSTM is shown in Figure 1 and Figure 2. Figure 1 represents an LSTM network containing two hidden layers; for a single moment, it is a BP neural network, but the information from the hidden layers trained at T = 1 after unfolding along the time axis is passed on to the next moment T = 2. The horizontal line running through the whole figure in Figure 2 represents the state of the transmission unit, which ensures the invariance of the information transmission inside the unit by linear transformation, and it is the most core module in the long- and short-term memory network. In LSTM, the information about the state of the unit is screened by a threshold structure, which allows the selective passage of information. It consists of a sigmoid neural network layer and a two-by-two multiplication operation. Sigmoid is a non-linear activation function contained in a threshold structure. The output of the gate structure ranges from 0 to 1 and defines the degree to which the information passes through. The tanh layer in Figure 2 is an activation function that maps the actual input to the range [−1, 1].

(13) $$f_{u}=sigmoid\left(W_{f}x_{t}+W_{Hf}H_{t-1}+b_{f}\right)$$

(14) $$i_{u}=sigmoid\left(W_{i}x_{t}+W_{Hi}H_{t-1}+b_{i}\right)$$

(15) $$o_{u}=sigmoid\left(W_{o}x_{t}+W_{Ho}H_{t-1}+b_{o}\right)$$

(16) $$c_{t}=c_{t-1}⊗{\left(f_{u}\right)}_{t}+{\left(i_{u}\right)}_{t}⊗\left(\tanh\left(W_{c}x_{t}+W_{Hc}H_{t-1}+b_{c}\right)\right)$$

(17) $$H_{t}=o_{t}⊗\tanh\left(c_{t-1}\right)$$

In Equations (13)–(16), $H_{t-1}$ denotes the previous hidden unit states, which are combined with the weights of the three units by elementwise addition to obtain the current unit state $c_{t}$ . Element-by-element multiplications between the input units, unit states, and output units of the hidden layer are denoted by symbols. The kernel function is represented using tanh and sigmoid functions. Equations (9)–(14) denote the computation of oblivion, input, and output cells, respectively. Equations (16) and (17) represent the current state of the memory and hidden units at time step *t*.

**Figure 3:**
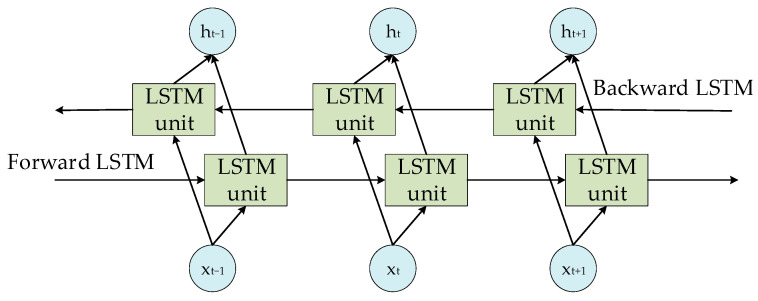
Structure of BiLSTM model.

BiLSTM addresses the unidirectional sequence processing limitations of the conventional LSTM by handling inverse dependencies. This set-up, as depicted in Figure 3, includes an added inverse LSTM layer, which aids in capturing different features in the sequence. This enhancement significantly reduces the chances of gradient vanishing or exploding—a common issue in unidirectional LSTM. As a result, BiLSTM tends to perform better than LSTM in a variety of long time sequence data processing tasks.

### 2.5. Offshore Power Prediction Model Based on PCA-VMD-SSA-BiLSTM

**Figure 4:**
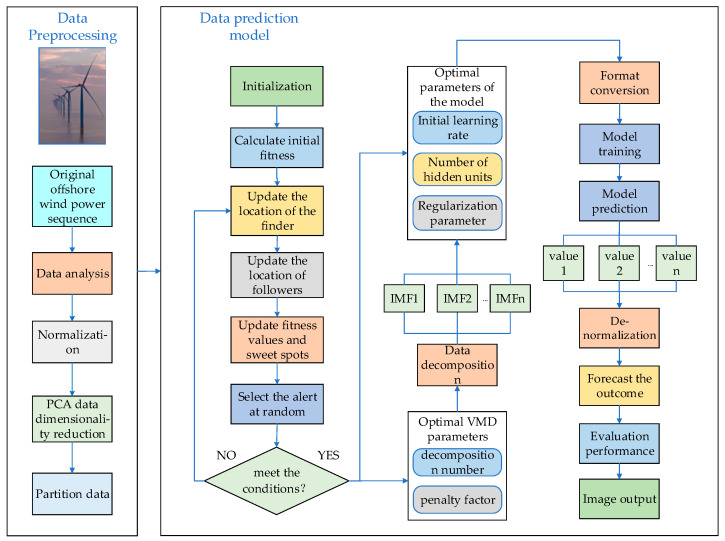
Structure of the combined PCA-SSA-VMD-BiLSTM prediction model.

This paper presents a multi-algorithm optimization model based on the BiLSTM network, capable of effectively learning and training time series data to extract time dimension features. The model, as visualized in Figure 4 and Algorithm 1, harnesses the valuable information contained within the historical time series of offshore wind power, reflecting ultra-short-term wind power fluctuations. The model framework unfolds in three interconnected stages. First, PCA is used for a pre-processing operation on the data, and the K and α values of VMD are optimized using the SSA algorithm. This leads to the model being divided into k sub-prediction modules using the optimized VMD algorithm. In the second stage, we construct and optimize the SSA-BiLSTM neural network with the number of hidden units, learning rate, and the regularization parameter of BiLSTM. For the final stage, the sub-prediction modules are unified and superimposed, following which the outputs undergo back-normalization, among other operations, and are subsequently evaluated.
**Algorithm 1:** PCA-SSA-VMD-BiLSTM Offshore-Wind-Power-Prediction AlgorithmInput:Environmental factors: wind speed, wind direction, humidity, temperature, pressureInitialization parameters: population size, number of iterations, number of modal decompositions, penalty coefficients, neural network model parametersOutput: Forecast results, model evaluation indicators (RMSE, MAE, MAPE, R^2^)(1)Data cleaning: Cleaning of wind series and environmental factor series data. “Bad data” due to observation errors, communication failures, etc., are eliminated on a daily basis.(2)The cleaned data series are downscaled by principal component analysis (PCA), and the key series affecting wind power are filtered with the threshold of 85% cumulative contribution rate to eliminate the redundancy of different time series data.(3)Optimizing the VMD by the SSA algorithm, combining the features of fast solving speed and high accuracy of SSA, and selecting the average envelope entropy as the adaptability function to adaptively determine the best selected parameters k and α of the VMD.(4)Decompose the environmental factor sequence into inherent modal components IMF1, IMF2, …, IMFn at different frequencies by the optimized VMD method in step 3, and decompose the different scale fluctuations and trends existing in the original environmental signal step by step.(5)Optimization of BiLSTM by SSA algorithm, MSE is used as the fitness function of SSA to determine the optimal initial learning rate, the number of hidden units, and the regularization parameters of BiLSTM.(6)Converting the offshore wind power time data sequence obtained in step 4 into a format that makes it suitable for a dataset for training the BiLSTM network.(7)The training set of the dataset obtained in step 6 is input into the BiLSTM model for training until the target accuracy is reached.(8)After the model is trained, the training data are saved and input to the test set for testing.

## 3. Experiment and Analysis

### 3.1. Experimental Data

In this paper, the actual generating power data of a cluster of wind farms with a rated power of 200 MW are used as the experimental sample data, and the environmental factors include humidity, temperature, and barometric pressure, as well as the actual data of wind speed and direction at different heights, and the information of the data recorded at each time coincides with the time of the actual power output. From the wind farm data space, each time to the observation point recorded data interval is 15 min. The data are divided according to the quarters, with each quarter selecting 1500 sets of data from the datasets that have been cleansed of outliers for the experiment, with the first 80% serving as the training sample set and the last 20% serving as the prediction sample set. Some datasets are listed in Table 1.

### 3.2. Variable Correlation Analysis

Table 2 shows the correlation analysis between the natural factors of the wind farm (wind speed at each height of the turbine, wind direction at each height of the turbine, temperature, pressure, humidity) and the output power of the offshore wind turbine, and the correlation coefficients used are Pearson, Spearman, and Kendall.

Form Table 2, the correlation coefficients for wind speed at various heights range from 0.69 to 0.82, indicating a strong correlation with the wind turbine’s actual output power. This is because the magnitude of wind speed is a direct determinant of the turbine’s power generation capacity. In terms of wind direction, the coefficients vary from −0.17 to −0.23. The wind direction is crucial as it affects the angle of interaction between the airflow and the turbine blades. When the wind direction aligns with the blade rotation, it optimizes the conversion of kinetic energy into mechanical energy, thereby enhancing the efficiency of offshore power generation. The temperature coefficients lie between 0.11 and 0.25. Extreme temperatures can adversely affect the wind turbine’s performance: high temperatures may cause component expansion and deformation, while low temperatures can impair the lubrication of bearings and gears. The barometric pressure coefficients, ranging from −0.23 to −0.10, show a negative correlation with wind power output. Variations in barometric pressure influence wind speed; a significant pressure difference can increase wind speed, subsequently boosting power generation in offshore wind turbines.

Regarding humidity, the coefficients are around −0.15 and are also negatively correlated with power generation. Increased humidity leads to higher water vapor content in the air, reducing air density and, consequently, the power output of offshore wind turbines. Based on this analysis, wind speed, direction, temperature, pressure, and humidity at each altitude are identified as key input variables for the subsequent modeling process.

### 3.3. Wind Power Autocorrelation Analysis

**Figure 5:**
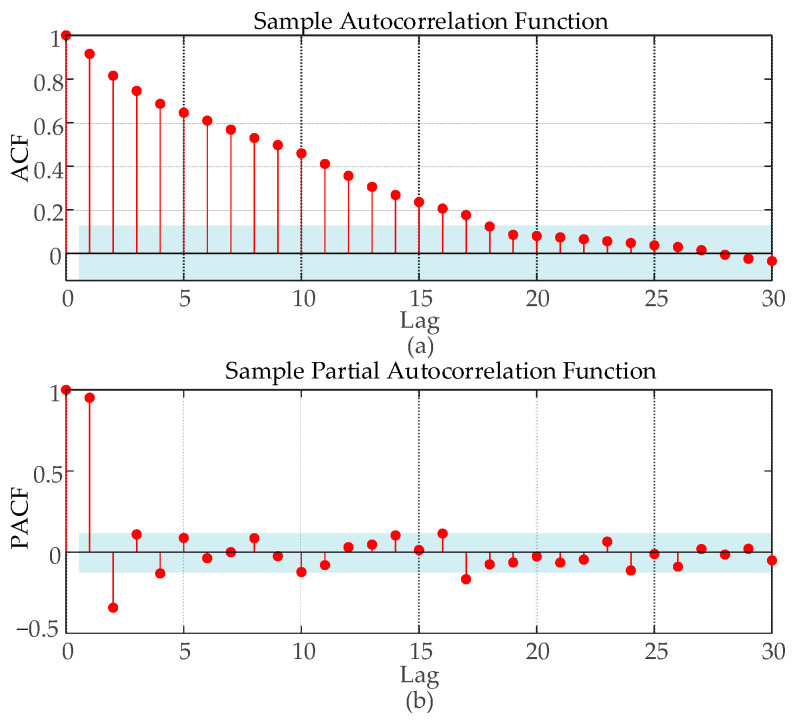
ACF and PACF diagrams of wind power. (**a**) Wind power autocorrelation diagram; (**b**) Wind power partial autocorrelation.

In this paper, a rolling modeling mechanism is used for prediction. To determine the time step setting of the prediction model, the autocorrelation function (ACF) and partial autocorrelation function (PACF) are used to analyze the autocorrelation step of the data used. As can be seen from Figure 5, in the wind power data interval, the ACF plot is characterized by a trailing tail and the PACF plot is characterized by a truncated tail, which satisfies the prediction characteristics of the regression model. As shown in Figure 5a, the ACF diagram enters the confidence interval with a lag of about 18 steps, so in the process of model training and prediction, the historical data of the first 18 groups (4.5 h) are selected as the characteristic attributes of the data for the next time node, and the model is built by predicting the offshore wind power values at the next time point (15 min) and by adding the actual values of the current prediction as the latest historical values for the next prediction.

### 3.4. Model Evaluation Indicators

In this paper, the Root Mean Square Error RMSE, Mean Absolute Error MAE, Mean Absolute Percentage Error MAPE, and Coefficient of Determination R^2^ are used as the model prediction accuracy evaluation indexes to assess all aspects of the involved methods, which are expressed as follows:

(1) Root Mean Square Error (RMSE) indicates the degree of deviation between the predicted and actual values of the model.
(18) $$\mathrm{RMSE}=\sqrt{\frac{1}{N}\sum_{t=1}^{N}{\left(u_{actual}^{t}-u_{predict}^{t}\right)}^{2}}$$
(2) Mean Absolute Error (MAE) reflects the reality of the error, and the value becomes larger when the error is larger.
(19) $$\mathrm{MAE}=\frac{1}{N}\sum_{t=1}^{N}\left|u_{actual}^{t}-u_{predict}^{t}\right|$$
(3) Mean Absolute Percentile Error (MAPE) is used to measure forecast accuracy. Smaller MAPE values indicate that the model is more accurate in forecasting.
(20) $$\mathrm{MAPE}=\frac{1}{N}\sum_{t=1}^{N}\left|\frac{u_{actual}^{t}-u_{predict}^{t}}{u_{actual}^{t}}\right|$$
(4) R^2^ (R-squared, R^2^) represents the percentage of variance of the dependent variable in the model that can be explained by the independent variable, with a higher R^2^ indicating that the model explains more of the variability.
(21) $$R^{2}=1-\frac{\sum_{t=1}^{N}{\left(u_{predict}^{t}-{\bar{u}}_{actual}^{t}\right)}^{2}}{\sum_{t=1}^{N}{\left(u_{acutal}^{t}-{\bar{u}}_{actual}^{t}\right)}^{2}}$$

### 3.5. Data Pre-Processing

The raw data contain wind speed, wind direction, pressure, temperature, humidity, etc. In order to reduce the redundancy and correlation of the feature sequences, the PCA method is used to determine the minimum number of variables required and to analyze the multivariate predictors. First, the data were normalized to unify the magnitude of each parameter, and then component extraction was performed to calculate the covariance matrix of the normalized training data, to find the eigen root and contribution rate of the covariance matrix, and to extract the principal components based on the cumulative contribution rate. The calculated principal component eigenvalues and cumulative contribution rates are shown in Table 3, which displays the principal component eigenvalues and cumulative contribution. As can be seen from Table 3, The cumulative contribution η of the first four feature sequences is close to 85%. The filtered principal components can better represent the original feature sequences and have high information synthesis ability. Therefore, the first three feature sequences are selected to replace the original input sequences.

### 3.6. SSA-VMD Model

**Figure 6:**
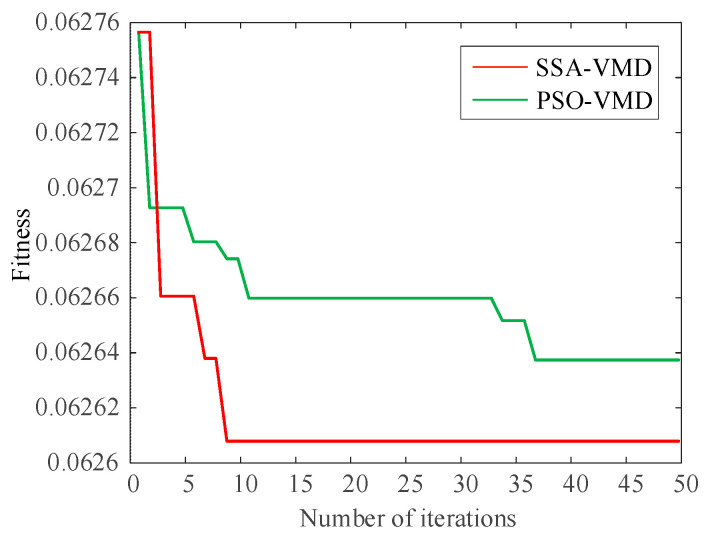
Comparison of fitness curves of different algorithms.

**Figure 7:**
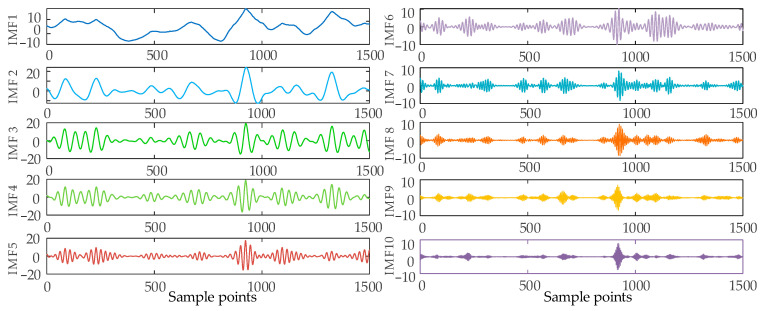
Wind power time domain in the first quarter based on SSA-VMD decomposition.

**Figure 8:**
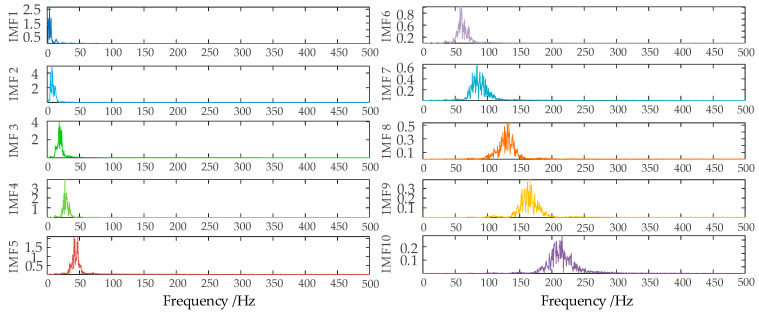
Spectrogram of data decomposition.

In order to ensure the quality of the input data and eliminate the influence of noise on the prediction, this paper decomposes the wind power input sequence by the VMD algorithm optimized by the SSA algorithm. Take the first quarter as an example. Firstly, the number of sparrow population is set to 30, the maximum number of iterations is 50, the number of variables is 2, the range of K value is [2, 10], the range of penalty factor is [500, 3000], the initial warning value is set to 0.6, the proportion of discoverers is 0.7, and the rest are joiners; then, SSA is used to optimize the VMD parameters. The iterative evolution results of the SSA algorithm are shown in the following figure. From Figure 6, it can be seen that the sparrow algorithm is gradually stabilized after the 8th generation of evolution, and the value of the optimal fitness function is 0.06261. The computational results show that the SSA algorithm’s solution speed and computational accuracy are better than the PSO algorithm. The optimal k and α values of VMD obtained by iterative calculation of SSA algorithm are [10, 2930]. Figure 7 shows the time-domain waveforms of the 10 IMFs decomposed by PCA-SSA-VMD. The spectrum obtained by performing Hilbert transform on the IMFs is shown in Figure 8. From the figure, it can be seen that the eigenfrequencies of the IMFs in different time domains are clearly distinguished and more regularly distributed, which effectively avoids the problem of modal aliasing.

### 3.7. Ultra-Short-Term Offshore-Wind-Power-Prediction Results Based on PCA-SSA-VMD-BiLSTM

BiLSTM prediction models are devised for each of the ten IMF sequences procured from the SSA-VMD decomposition, incorporating ‘adam’ as the model’s optimizer. The learning rate decreasing factor bears the value of 0.2, supplementing an L2 regularization methodology to avert overfitting and to enhance the model’s generalization capacity. The optimization ambit for the regularization parameter is established between 0.0001 and 0.01. The optimal values for the initial learning rate, the quantity of hidden units, and the regularization parameters are obtained from the SSA algorithm. Consequently, the optimal initial learning rate is 0.01, the optimal number of hidden units is represented as [19, 30, 72], and the regularization parameter is 0.0002.

**Figure 9:**
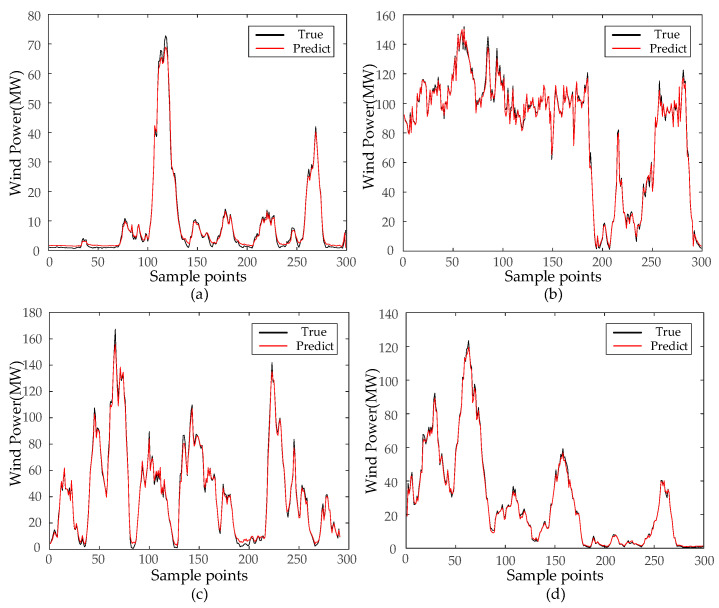
Results of four quarterly model projections. (**a**) Forecast results for the first quarter; (**b**) Forecast results for the second quarter; (**c**) Forecast results for the third quarter; (**d**) Forecast results for the fourth quarter.

Each IMF’s prediction results are consolidated to derive the wind-power-prediction output values. As demonstrated in Figure 9, the PCA-SSA-VMD-BiLSTM model suggested in this research paper exhibits commendable training precision in offshore wind-power-prediction application, sustaining considerable stability amidst fluctuating offshore wind power data. Upon calculating each model’s evaluation index, the R^2^ reads 0.9954, nearing a value of 1. The RMSE, MAE, and MAPE values are recorded as 0.9741 MW, 0.7536 MW, and 2.76%, respectively.

From Table 4, it can be seen that the RMSE ranges from 0.9741 to 2.4504, where the evaluation metrics cross over in the second and third quarters due to the drastic and stochastic climate change in the second and third quarters, which results in lower precision than the other two quarters.

### 3.8. Multi-Model Comparison

**Figure 10:**
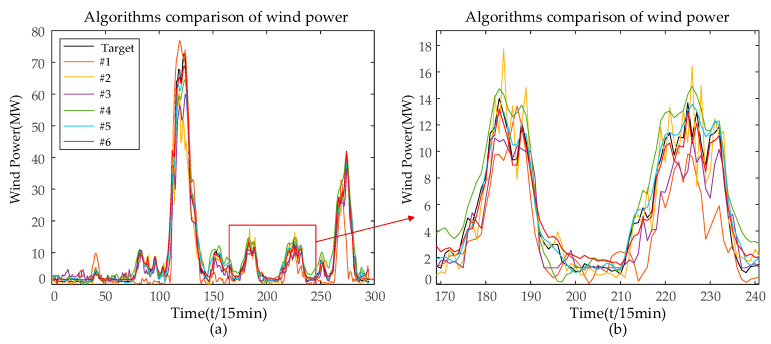
(**a**) The predictions results of several models (**b**) Partial enlarged view on the horizontal axis [170, 240].

**Figure 11:**
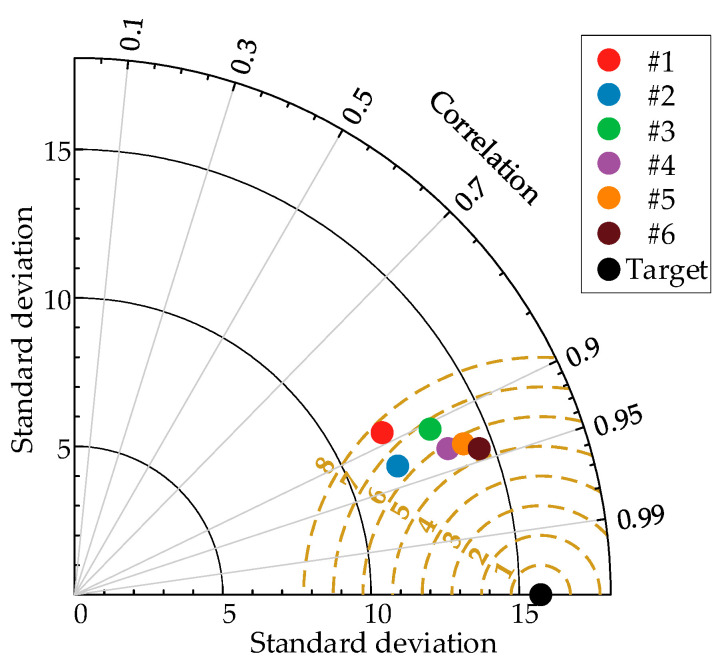
Taylor Diagram: Actual and Prediction models.

In order to verify the superiority of the PCA-VMD-SSA-BiLSTM wind-power-prediction model proposed in this paper, as well as to show that the combined prediction model proposed in this paper can significantly enhance and improve the accuracy of the wind power prediction, simulation studies have been carried out on the BP, LSTM, BiLSTM, SSA-BiLSTM, and VMD-BiLSTM models after data pre-processing, respectively, and the results are shown as follows for the first quarter as an example. In Figure 10, the black line represents the actual values, while the remaining six colored lines represent the predicted values, and the closer their positions are to the black line, the higher the prediction accuracy of the model. In the legend, #1 represents PCA-BP, #2 represents PCA-LSTM, #3 represents PCA-BiLSTM, #4 represents PCA-SSA-BiLSTM, #5 represents PCA-VMD-BiLSTM, and #6 represents PCA-SSA-VMD-BiLSTM. It can be observed that the original sequence is more volatile, with a larger gap between the peaks and the valleys, but the overall error of the prediction of each model is small, and it can basically reflect the trend of the sequence, whereas the line of the predicted values of the PCA-SSA-VMD-BiLSTM model is closer to the black line, which indicates that the prediction accuracy of the PCA-SSA-VMD-BiLSTM is higher. Figure 11 uses a Taylor diagram. It can be seen that the model proposed in this paper has higher correlation than other methods. Furthermore, the distribution predicted by PCA-SSA-VMD-BiLSTM is closer to the observed target value than by other methods.

**Figure 12:**
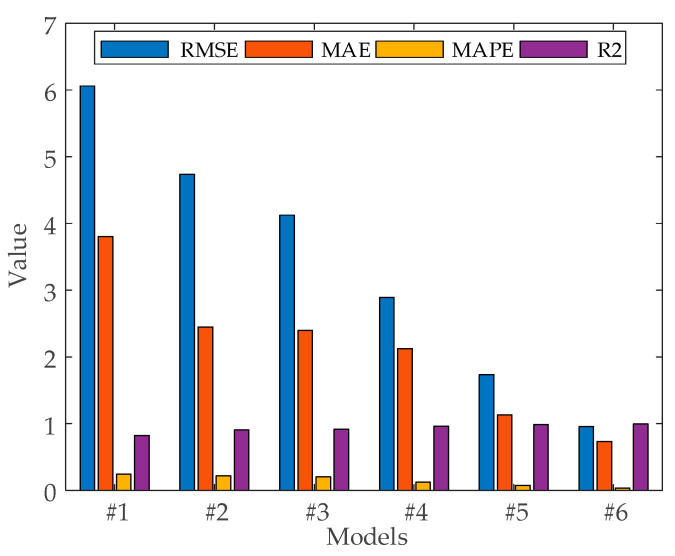
Error evaluation and evaluation coefficient results of different models.

Based on the prediction results in Figure 12 and Table 5, this paper analyzes the prediction effect of the model from different perspectives.

Comparison 1: The evaluation metrics show that BiLSTM reduces the prediction error RMSE by 1.9353 and 0.6108 MW, R^2^ improves by 0.0943 and 0.0141 MW, and MAPE improves by 0.0943 and 0.0141 MW, as compared to #1, #2, and #3 because of the model advantage of having a bidirectional logic gate structure. The results show that using the BiLSTM neural network model has better fitting ability and better accuracy than BP and LSTM neural network models for offshore wind power prediction. The model performance is BP < LSTM < BiLSTM.

Comparison 2: The evaluation metrics show that the combined model with the addition of optimization algorithms for optimization search of hyperparameters possesses higher accuracy compared to the single neural network model as compared to #3 and #4. The prediction error RMSE is reduced by 1.2336 MW, MAE is reduced by 0.9909 MW, and MAPE is reduced by 7.6605%. SSA mimics the searching behavior of sparrows in nature and has better robustness when dealing with complex, non-linear, or multimodal optimization problems.

Comparison 3: The evaluation metrics show that VMD is used to decompose and denoise the raw sequence data compared to #3 and #5, resulting in a reduction in RMSE by 2.3881 MW, a reduction in MAE by 1.2644 MW, a reduction in MAPE by 12.7115%, and an improvement in R^2^ by 0.0368. This indicates that decomposing and denoising the WT sequence can provide more effective information and thus improve the prediction accuracy.

Comparison 4: The evaluation metrics show that compared with #4, #5, and #6, the prediction error RMSE of the proposed combination model in this paper is reduced by 1.9369 and 0.7824 MW, MAE is improved by 1.3894 and 0.3985 MW, and MAPE is reduced by 8.909% and 3.858%. This is because the SSA algorithm finds the optimal number of modes K, penalty factor α, optimal training period, optimal initial learning rate, and optimal number of hidden units in both VMD and BiLSTM. The results show that the proposed model #6 can make more accurate predictions than models #4 and #5.

## 4. Conclusions

The emergence of global warming and energy crises has propelled the development of global clean energy. Among these, the installed capacity of offshore wind power increased exponentially. However, the unpredictability and uncertainty of offshore wind power poses challenges to the safe and stable operation of the power system. Existing offshore-wind-power-prediction models boast low accuracy and stability. A combined offshore-wind-power-prediction method, based on PCA-SSA-VMD and BiLSTM, is proposed herein. This approach analyzes the five key environmental factors of offshore wind power: temperature, humidity, air pressure, wind direction, and wind speed. The conclusions drawn are as follows:

(1) Offshore wind power data usually contain multiple parameters, such as wind speed, wind direction, temperature, humidity, pressure, etc., and there may be correlations between these variables. The PCA algorithm can be used to transform these high-dimensional data into low-dimensional data, while retaining the main features of the data, which are linear combinations of the original features, to better reflect the main characteristics of the data, which can reduce the complexity of the data and improve the efficiency of the model calculation.
(2) The Bidirectional Long- and Short-Term Memory Network (BiLSTM) demonstrates a significant advantage over the Long- and Short-Term Memory Network (LSTM) as it incorporates both preceding and forthcoming data points of wind speed sequences. This feature enhances the accuracy of wind speed prediction. However, the BiLSTM model exhibits high sensitivity towards parameter choice. This paper employs the sparrow search algorithm for hyperparameter optimization of the model. Comparative experiments reveal superior accuracy when the SSA algorithm is integrated, validating the necessity and effectiveness of hyperparameter optimization using the SSA algorithm.
(3) The VMD decomposition algorithm can reduce the noise and eliminate the non-smoothness of the original wind power data. In order to solve the problem of selecting the optimal value of the quadratic penalty and factor and modal decomposition number in VMD, the SSA-VMD model is introduced, and the sparrow search algorithm (SSA) is used as an optimization algorithm to determine the optimal parameters in VMD, and the envelope spectral feature is used as a criterion for judging the goodness of the features extracted from the VMD as an adaptive function; the optimized post-variable modal decomposition (VMD) method is used with the SSA algorithm to wind power output time series decomposition and can effectively improve the model prediction performance.

In this paper, these results demonstrated the developmental potential and application value of the developed model. We will consider introducing more influencing factors to improve the prediction accuracy, for example, considering the influence of wind turbine location, the final prediction results will be different for different geographic locations of wind farms with different wind speeds, wind directions, and other meteorological data, which is the direction of our further research in the future.

## Figures and Tables

**Table 1:** Partial datasets of this experiment.

Time	10 m Wind Speed	30 m Wind Speed	50 m Wind Speed	10 m Wind	30 m Wind	50 m Wind	Environment Temperature	Air Pressure	Relative Humidity	Actual Generating Power
	(m/s)	(m/s)	(m/s)	Direction (°)	Direction (°)	Direction (°)		(hpa)		(MW)
0:00	2.54	2.79	2.03	169.86	167.79	161.09	−11.85	892.89	52.67	1.30
0:15	1.83	2.74	2.14	183.81	178.96	166.25	−11.29	892.83	51.02	1.23
0:30	2.64	3.03	2.39	160.37	186.14	163.76	−12.17	892.66	53.78	1.00
0:45	3.31	3.14	2.84	157.74	187.09	154.49	−13.10	892.57	57.13	0.95
1:00	3.32	3.01	2.49	163.4	186.17	174.76	−13.02	892.43	56.13	0.92
1:15	3.36	2.57	1.32	163.74	197.22	182.64	−13.30	892.3	57.48	0.91
1:30	3.16	2.45	1.37	164.08	198.79	191.16	−13.49	892.22	58.03	0.93

**Table 2:** Examples of selected environmental data.

Environmental Factor	Correlation Coefficient		
	Pearson	Spearman	Kendall
50 m wind speed	0.8167	0.8072	0.7659
30 m wind speed	0.7959	0.8322	0.7239
10 m wind speed	0.7867	0.8674	0.6944
50 m wind direction	−0.2067	−0.3625	−0.2312
50 m wind direction	−0.1959	−0.3540	−0.2238
10 wind direction	−0.1736	−0.3068	−0.2025
Temperature	0.2555	0.1506	0.1136
Pressure	−0.2349	−0.1240	−0.1075
humidity	−0.2437	−0.1690	−0.1491

**Table 3:** Principal component eigenvalues and cumulative contribution.

Principal Component Number	Characteristic Value	Variance Contribution %	Cumulative Contribution Rate %
Z_1_	2.8976	32.1954	32.1954
Z_2_	2.5399	28.2211	60.4165
Z_3_	1.3820	15.3662	75.7827
Z_4_	0.7526	8.3624	84.1454
Z_5_	0.7276	8.0848	92.2299
Z_6_	0.3381	3.7570	95.9869
Z_7_	0.1641	1.8232	97.8101
Z_8_	0.1209	1.3429	99.1530
Z_9_	0.0762	0.8469	99.9999

**Table 4:** Four quarterly projected evaluation indicators.

Season	RMSE/MW	MAE/MW	MAPE	R^2^
1st quarter	0.9741	0.7536	2.76%	0.9954
2nd quarter	2.1186	1.5256	1.61%	0.9969
3rd quarter	2.4504	1.8883	3.08%	0.9967
4th quarter	1.2447	0.8910	2.45%	0.9981

**Table 5:** Comparison of prediction errors.

Model	Abbreviation	MAPE	RMSE/MW	MAE/MW	R^2^
PCA-BP	#1	24.26%	6.0599	3.8046	0.8243
PCA-LSTM	#2	21.93%	4.7368	2.4501	0.9045
PCA-BiLSTM	#3	20.15%	4.1246	2.3975	0.9186
PCA-SSA-BiLSTM	#4	12.49%	2.8910	2.1240	0.9601
PCA-VMD-BiLSTM	#5	7.44%	1.7365	1.1331	0.9857
PCA-SSA-VMD-BiLSTM	#6	3.58%	0.9541	0.7346	0.9957

## Data Availability

The data presented in this study are available on request from the corresponding author.
